# Structural analysis of spike proteins from SARS-CoV-2 variants of concern highlighting their functional alterations

**DOI:** 10.2217/fvl-2022-0003

**Published:** 2022-08-02

**Authors:** Kundan Solanki, Sajjan Rajpoot, Ashutosh Kumar, Kam Y J Zhang, Tomokazu Ohishi, Nik Hirani, Khandu Wadhonkar, Pramod Patidar, Qiuwei Pan, Mirza S Baig

**Affiliations:** ^1^Department of Biosciences & Biomedical Engineering, Indian Institute of Technology Indore, Simrol, Indore, 453552, India; ^2^Laboratory for Structural Bioinformatics, Center for Biosystems Dynamics Research, RIKEN, Tsurumi, Yokohama, Kanagawa, 230-0045, Japan; ^3^Institute of Microbial Chemistry, Microbial Chemistry Research Foundation, Numazu-Shi, Shizuoka, 410-0301, Japan; ^4^MRC Centre for Inflammation Research, Queen's Medical Research Institute, University of Edinburgh, Edinburgh, EH164TJ, UK; ^5^Department of Gastroenterology & Hepatology, Erasmus MC-University Medical Center, Rotterdam, The Netherlands

**Keywords:** ACE2, Delta, monoclonal antibodies, mutations, Omicron, RBD, SARS-CoV-2, spike

## Abstract

**Aim:** Mutations in the SARS-CoV-2 spike (S) protein have dramatically changed the transmissibility and pathogenicity of the virus. Therefore, we studied the binding affinity of Omicron spike-receptor binding domain (S-RBD) with human ACE2 receptor. **Materials & methods:** We used pyDockWEB and HADDOCK 2.4 docking for our study. **Results:** Computational docking indicated higher binding affinity of Omicron S-RBD as compared with wild-type SARS-CoV-2 and Delta S-RBD with ACE2. Interface analysis suggested four mutated residues of Omicron S-RBD for its enhanced binding. We also showed decreased binding affinity of Omicron and Delta S-RBDs with monoclonal antibodies. **Conclusion:** Compared with wild-type SARS-CoV-2, Omicron S-RBD exhibit higher binding with ACE2 and lower affinity against monoclonal antibodies.

SARS-CoV-2 is an enveloped positive-sense single-stranded RNA virus with a genome size of 29.9 kb [1]. It contains several structural and non-structural proteins responsible for its life cycle. The Spike glycoprotein (S protein) is responsible for the binding of the virus to the host ACE2 receptor [2]. The S protein contains S1 and S2 subunits, of which receptor-binding domain (RBD) present in the S1 subunit is specifically involved in binding the virus with the host ACE2 receptor. This makes the S-RBD region of the virus an essential therapeutic and preventive target for curbing the rate of infection [3]. Indeed, mutations in the S-RBD regions are responsible for the emergence of novel variants such as B.1.1.7 (Alpha), B.1.351 (Beta), P.1 (Gamma), B.1.617.1 (Kappa) and B.1.617.2 (Delta) which enhances the binding affinity and infectivity of the virus compared with its wild-type counterpart [4]. In terms of infectivity, several studies have reported that the variant of concern (VOCs) strains of SARS-CoV-2 (i.e., Alpha, Beta, Gamma and Delta) have 45–71% more transmissibility than the wild-type SARS-CoV-2 [5,6]. In addition, the meta-analysis of patients infected by the VOCs strains conducted from June 2020 to October 2021, found increased susceptibility of the affected patient to hospitalization, intensive care unit admissions and death, which suggests the greatly increased pathogenic nature of VOCs strains compared with its wild-type counterpart [7,8]. Several mutations in the non-RBD regions of the VOC strains, such as D614G (present in Alpha, Beta, Gamma, Delta and Omicron) and in RBD regions, such as T478K (present in Delta and Omicron), N501Y (present in Alpha, Beta, Gamma and Omicron), K417N/T and E484K/A (present in Beta, Gamma and Omicron) play a significant role in enhancing the pathogenicity and infectivity of the virus [9–12]. As most of the mutations in the S-RBD region play an important role in its infectivity and pathogenicity, therapeutic strategies targeting the S-RBD region, such as designing a novel peptide inhibitor, have been explored [13–15]. Such an approach can also be employed in the case of Omicron.

On 24 November 2021, South Africa reported the identification of a new SARS-CoV-2 variant, Omicron (B.1.1.529), to the WHO. On 26 November 2021, WHO designated the Omicron (B.1.1.529) as a VOC, based on rapidly increasing numbers of infections of the variant in several parts of the world [16]. Phylogenetic analysis shows Omicron forms a new monophyletic clade distant from other SARS-CoV-2 variants. Based on the percentage of sequence identity, it was revealed that Omicron shows a close relationship with the Alpha variant, and the relationship order was Alpha, Gamma, Delta, Beta, Mu and wild-type SARS-CoV-2 [17]. There are several substitutions in the spike protein of Omicron. Some are known substitutions, as seen in previous VOC strains, which were involved in reduced susceptibility to monoclonal antibody therapy or reduced neutralization to convalescent sera [18]. As far as disease severity is concerned, infection with Omicron have led to lower disease severity compared with Delta. This can be attributed to changes in the virus that limits its spread in the lungs and also due to increased immunity in the population infected with SARS-CoV-2 or due to mass immunization [19–21]. Although, the hospitalization rate in children/adolescents (aged 6–18 years) were higher, the fatality ratio was lower in Omicron than Delta, suggesting its less severity in children [22,23]. Studies on the neutralizing activity against Omicron have found lower neutralizing activity from the sera of convalescent and double-vaccinated candidates while individuals exposed three to four-times with spike protein in addition to vaccination showed better activity [24,25]. This data suggests that promoting affinity maturation of antibodies in individuals who have already been infected or vaccinated would provide an additional protection against infection from the virus. In agreement with previous studies discussing the SARS-CoV-2 spike protein mutations [26–28], we performed the sequence analysis of each VOC, including Omicron, for comparison of spike protein mutations with wild-type SARS-CoV-2 through the Global initiative on sharing all influenza data (GISAID) CoVsurver: Mutation Analysis of hCoV-19 application (https://www.gisaid.org/epiflu-applications/covsurver-mutations-app/) (Table 1). In comparison with the wild-type of SARS-CoV-2 and other VOC (Alpha, Beta, Gamma and Delta), the spike protein of Omicron consists of the highest number of mutations (total of 42 amino acid changes), including 35 amino acid substitutions, six deletions and one insertion (Table 1). Out of 42 amino acid changes, 15 substitutions are in the RBD of the spike [29], as indicated in Table 1. As discussed, the mutations in the RBD are critical as they severely impact the binding of spike protein with the ACE2 receptor [30]. For example, a few common substitutions among these VOCs, like N501Y and K417N, have already been shown to enhance the binding of the virus with the host receptor resulting in their increased transmissibility and antigenic escape [15,31]. Thus, it becomes imperative to dissect the critical mutated residues in the S-RBD of the Omicron which helps in mediating the enhanced binding of the virus to the human ACE2 receptor. The Delta variant of SARS-CoV-2 was found to have increased transmissibility and infectivity due to enhanced binding of its S-RBD with the ACE2 receptor [32]. Moreover, a study has also found enhanced binding of S-RBD of other SARS-CoV-2 VOC strains including Delta with the ACE2 receptor [15]. Thus, the current study aims to computationally access the binding affinity of Omicron S-RBD with the human ACE2 receptor and compare it with the binding affinity of the S-RBD of the wild-type SARS-CoV-2 and with the Delta variant to the ACE2 receptor to evaluate the infectivity of the Omicron. It also aims to dissect the critical mutated residues in the S-RBD region of the Omicron involved in its binding to the ACE2 receptor. Furthermore, docking of S-RBD of variants with three monoclonal antibodies (mAbs) were conducted to check the binding affinity and neutralization capacity of those antibodies against the variants in comparison to the wild-type SARS-CoV-2 S-RBD.

**Table 1.** List of mutations in spike (S) protein sequence of SARS-CoV-2 variants of concern in comparison with the wild-type Wuhan Spike hCoV-19/Wuhan/WIV04/2019 sequence obtained through Global initiative on sharing all influenza data CoVsurver: mutation analysis of hCoV-19 application.

No.	Variants		NCBI accession ID	Spike protein mutations	Identity with WT (%)	AA change	WHO status of VOC, date
1	Spike hCoV-19/Wuhan/WIV04/2019 (wild-type)		P0DTC2/ QHR63260	–	–	–	–
2	Alpha	B.1.1.7	7FET_A	H69**^del^**, V70^del^, Y144**^del^**, **N501Y**, A570D, D614G, P681H, R682G, R683S, R685S, T716I, S982A, K986P, V987P, D1118H	99	15	Previous VOC 9 March 2022
3	Beta	B.1.351	7V8C_A	L18F, D80A, D215G, L242**^del^**, A243**^del^**, L244**^del^**, R246I, **K417N, E484K, N501Y**, D614G, A701V, Y1209E, I1210F, K1211G, W1212S, P1213G, W1214G	98.8	18	Previous VOC 9 March 2022
4	Gamma	P.1	7V84_A	L18F, T20N, P26S, D138Y, R190S, **K417T, E484K, N501Y**, D614G, H655Y, R682G, R683S, R685S, K986P, V987P, T1027I, Y1209E, I1210F, K1211G, W1212S, P1213G, W1214G	98.2	22	Previous VOC 9 March 2022
5	Delta	B.1.617.2	7TPH_C	T19R, G142D, E156G, F157**^del^**, R158**^del^**, **L452R, T478K**, D614G, P681R, R682G, R683S, R685S, D950N	99.1	13	Current VOC 11 May 2021
6	Omicron	B.1.1.592	7QO9_A	A67V, H69**^del^**, V70**^del^**, T95I, G142D, V143**^del^**, Y144**^del^**, Y145**^del^**, N211**^del^**, L212I, **^ins^**214EPE, **G339D, S371L, S373P, S375F, K417N, N440K, G446S, S477N, T478K, E484A, Q493R, G496S, Q498R, N501Y, Y505H**, T547K, D614G, H655Y, N679K, P681H, R682G, R683S, R685S, N764K, D796Y, N856K, Q954H, N969K, L981F, K986P, V987P	97.1	42	Current VOC 26 November 2021

The mutations in spike protein RBD are highlighted in bold.

AA: Amino acid; del: Deletion; ins: Insertion; RBD: Receptor binding domain; VOC: Variant of concern; WT: Wild-type.

## Materials & methods

### Structure retrieval, molecular docking & analysis of interacting residues

Studying the affinity of the Spike RBD with the human ACE2 receptor help in deciphering the infectivity of the virus [33]. Crystal structure of SARS-CoV-2-ACE2 complex (PDB ID: 6M17) was taken from RCSB PDB (https://www.rcsb.org/). Spike RBD of Omicron and Delta were prepared by deleting ACE2 from the crystal structure, removing water molecules and heteroatoms and adding polar hydrogens in Discovery Studio Visualizer (https://www.3ds.com/products-services/biovia/products/molecular-modeling-simulation/biovia-discovery-studio/). Further, site-specific mutations on RBD specific to Omicron and Delta as indicated in Table 1 were created in Discovery Studio Visualizer. For the preparation of the ACE2 receptor, a similar strategy was employed by deleting the Spike RBD structure and adding polar hydrogen atoms in Discovery Studio Visualizer. The prepared structures were docked in pyDockWEB (https://life.bsc.es/pid/pydockweb) and HADDOCK 2.4 (https://wenmr.science.uu.nl/haddock2.4/) for studying the binding affinity of the Spike-RBD with the receptor. For comparison, Spike RBD of SARS-CoV-2 (PDB 6M17) was taken and was docked with ACE2 on the same platforms to compare the dock scores with the variant. The represented structures were prepared through the Discovery studio visualizer tool. Furthermore, 3Å interacting residues between the docked complex were analyzed in UCSF Chimera (https://www.rbvi.ucsf.edu/chimera) to deduce the important residues interacting between the complex.

### Docking studies of mAbs with spike RBD

To deduce the effect of currently available mAbs against the S-RBD of Omicron and Delta variant, docking of the Fab region of the three mAbs (casirivimab, bamlanivimab and etesevimab) on the S-RBD region of the virus were conducted. Crystal structure of the S-RBD of Omicron (PDB 7T9K) and Delta (PDB 7W92) were docked on three antibodies (casirivimab [PDB 6XDG], bamlanivimab [PDB 7KMI] and etesevimab [PDB 7F7E]) in pyDockWEB and HDOCK server. To compare the docked scores between the antibodies and the variants, docking of mAbs were further performed with the crystal structure of wild-type S-RBD already associated with the individual antibodies (PDB 6XDG, 7KMI and 7F7E) on the same platforms. All the representative docking structures were prepared using Discovery Studio Visualizer.

## Results

### Mutations in spike proteins of SARS-CoV-2 VOC increases their binding to ACE2 receptor on host cells

**Figure 1.**
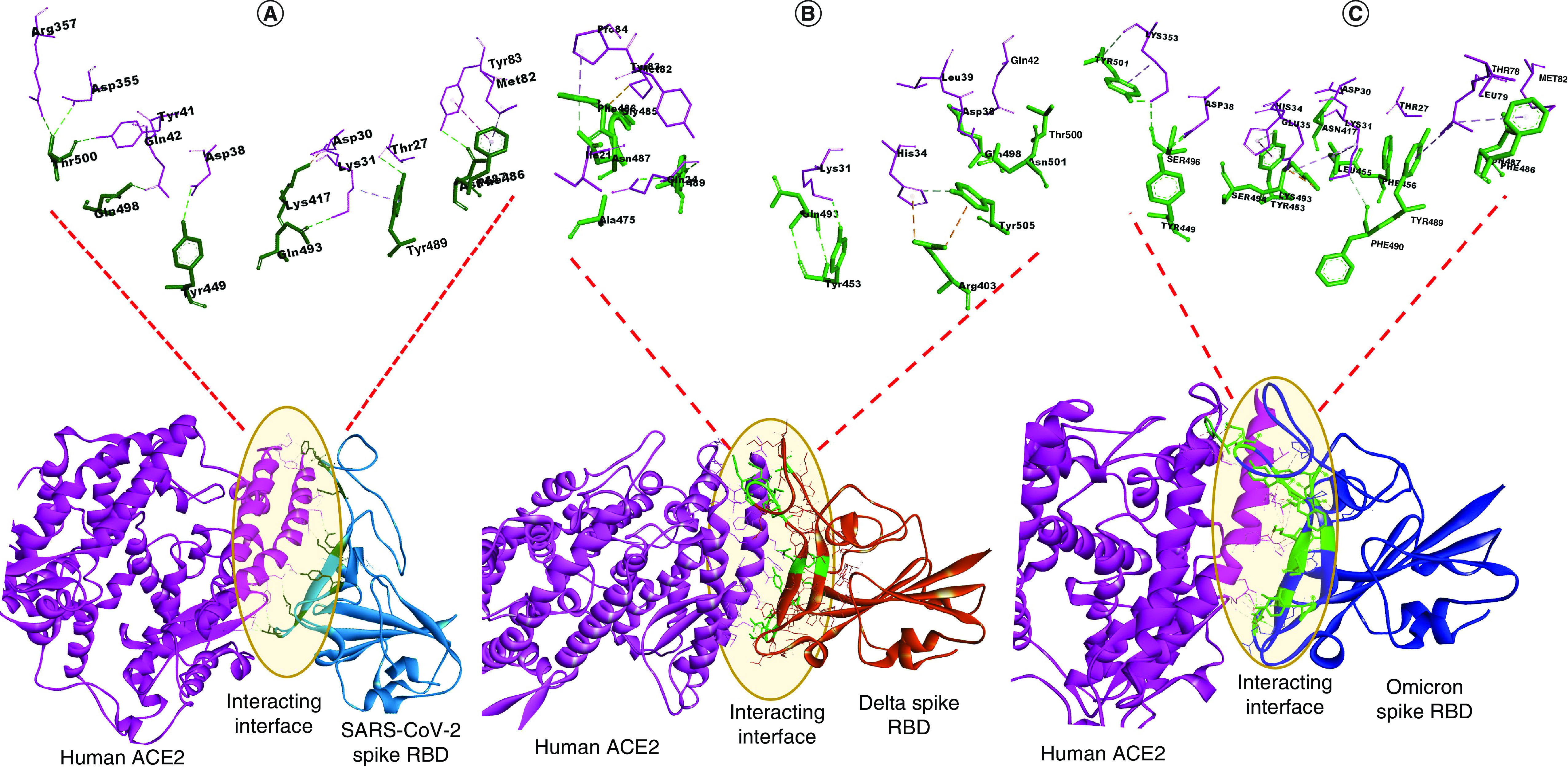
Docking of SARS-CoV-2, Delta and Omicron Spike receptor binding domain with ACE2 receptor. **(A)** Interface residues between docked complex of SARS-CoV-2 Spike RBD and ACE2 receptor. **(B)** Interface residues between docked complex of Delta Spike RBD and ACE2 receptor. **(C)** Interface residues between docked complex of Omicron Spike RBD and ACE2 receptor. SARS-CoV-2 Spike RBD is shown in light blue; Delta Spike RBD is shown in orange; Omicron Spike RBD is shown in dark blue; ACE2 is shown in pink. Interface residues between docked complex of Spike RBD and ACE2 are highlighted in green. RBD: Receptor binding domain.

The crystal structure of SARS-CoV-2 Spike RBD and ACE2 interaction (PDB 6M17) was used as the template for our study. We analyzed and obtained the interacting residues of the complex which served as the interface residue for further interaction study between the Omicron and Delta variant with the ACE2. The *in silico* mutated Omicron and Delta S-RBD were subjected to a molecular docking study with ACE2 in two protein–protein docking tools. The results indicated a significant increase in the dock score of both Omicron and Delta S-RBD with the ACE2 receptor on both the platforms (Table 2) suggesting an increased affinity of the S-RBD of variants with the ACE2 compared with that of the S-RBD of wild-type SARS-CoV-2. The representative docking interface have been shown in Figure 1. To obtain the dock score for comparison as well as to confirm the docking accuracy in accordance with the crystal structure complex, we performed the docking of SARS-CoV-2 S-RBD and ACE2. Study of 3Å interacting residues in UCSF Chimera (https://www.rbvi.ucsf.edu/chimera) between the complex suggested that out of 15 mutations in Omicron Spike-RBD, four mutated residues (K417N, Q493R, G496S and N501Y) showed interactions with the ACE2 receptor (Table 3), suggesting that mutation indeed elevated the binding affinity of the S-RBD with the receptor. Hence, it would be interesting to find the significance of these four residues by *in vitro* site-directed mutagenesis study to confirm whether this specific mutation helps in the increased affinity of the Omicron S-RBD with the ACE2. Although analysis of 3Å interacting residues between S-RBD of Delta and ACE2 did not show any mutated residues of Delta S-RBD (i.e., L452R and T478K) interacting with the ACE2 (Table 3), other residues of Delta spike RBD involved in the interaction with the ACE2 might play a significant role in its enhanced binding. However, this preliminary study indeed confirms the higher binding affinity of Omicron with host ACE2 receptor compared with the Delta and wild-type SARS-CoV-2 and can pose a potential threat to high rate of infection and rapid transmission.

**Table 2.** Dock scores of SARS-CoV-2, Delta and Omicron S-RBD with ACE2 receptor.

No.	Docking proteins		Docking score	
	SARS-CoV-2 spike RBD	Host receptor	pyDockWEB	HADDOCK 2.4
1	Wild-type spike RBD	ACE2	-76.09	-102 +/- 7.5
2	Delta spike RBD	ACE2	-79.35	-121.1 +/- 5.1
3	Omicron spike RBD	ACE2	-87.89	-147.7 +/- 10.7

RBD: Receptor binding domain.

**Table 3.** Interacting residues between wild-type and variant strains of SARS-CoV-2 S-RBD and hACE-2 within 3Å region.

Interacting proteins	Variants		
	SARS-CoV-2	Delta	Omicron
Spike-RBD residues	R403, Y453, A475, G485, F486, N487, C488, Y489, Q493, Q498, T500, N501, Y505	R403, Y453, A475, G485, F486, N487, C488, Y489, Q493, Q498, T500, N501, Y505	**N417**, Y449, Y453, L455, F456, F486, N487, Y489, F490, **R493**, S494, **S496**, **Y501**
ACE2 residues	I21, Q24, K31, H34, D38, L39, Q42, M82, Y83, P84, E87	I21, Q24, K31, H34, D38, L39, Q42, M82, Y83, P84, E87	T27, F28, D30, K31, H34, E35, D38, T78, L79, M82, K353

Bold residues indicate the mutated residues binding with ACE2 receptor.

ACE2: Angiotensin converting enzyme 2; RBD: Receptor binding domain.

### Decreased neutralization of mAbs to the S-RBD of variants compared with wild-type SARS-CoV-2

**Figure 2.**
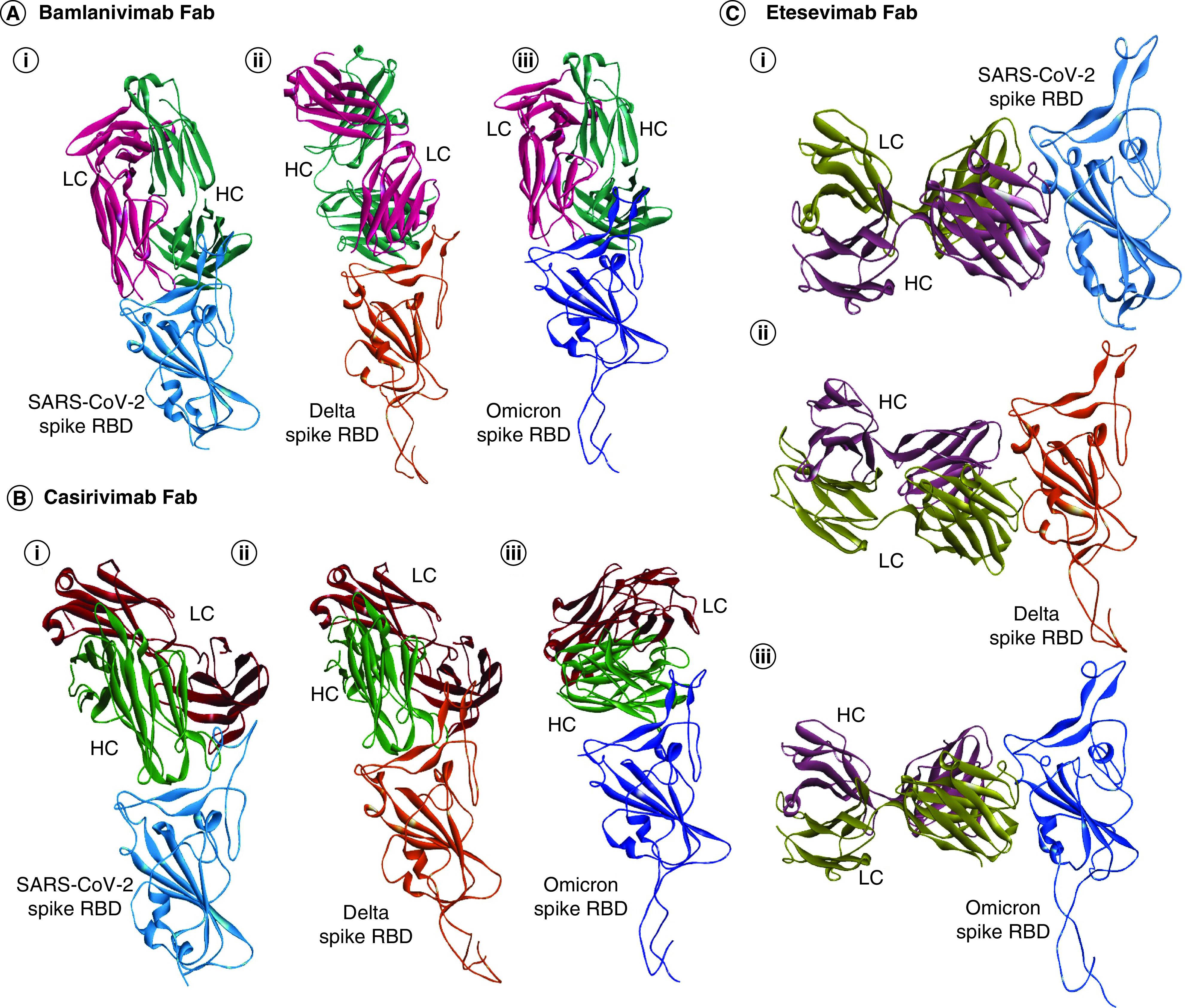
Docking of Spike receptor binding domain of SARS-CoV-2, Delta and Omicron with mAbs. **(A)** pyDockWEB analysis of Bamlanivimab Fab (HC&LC) against S-RBD of **(Ai)** SARS-CoV-2, **(Aii)** Delta and **(Aiii)** Omicron. **(B)** pyDockWEB analysis of Casirivimab Fab (HC&LC) against S-RBD of **(Bi)** SARS-CoV-2, **(Bii)** Delta and **(Biii)** Omicron. **(C)** pyDockWEB analysis of Etesevimab Fab (HC&LC) against S-RBD of **(Ci)** SARS-CoV-2, **(Cii)** Delta and **(Ciii)** Omicron. SARS-CoV-2 Spike RBD is shown in light blue; Delta Spike RBD is shown in orange; Omicron spike RBD is shown in dark blue; bamlanivimab Fab heavy chain is shown in dark green; light chain is shown in light red; Casirivimab Fab heavy chain is shown in light green; light chain is shown in maroon; etesevimab Fab heavy chain is shown in violet; light chain is shown in dark yellow. Fab: Fragment antigen-binding region; HC: Heavy chain; LC: Light chain; RBD: Receptor binding domain.

To deduce the binding affinity and thus the neutralization capacity of the variants with the currently known mAbs, docking of the Fab region (heavy and light chain) of the three mAbs (casirivimab, bamlanivimab and etesevimab) were done on the S-RBD of wild-type SARS-CoV-2, Delta and Omicron. The results suggested an enhanced binding affinity of all three mAbs on the S-RBD of the wild-type SARS-CoV-2. In comparison, both Delta and Omicron S-RBD showed reduced binding affinity to the mAbs (Table 4, Figure 2 & Supplementary Figure 1). Thus, this data suggests that mutations in the S-RBD of the variants provide an advantage to the virus in decreasing the binding affinity to the mAbs as well as in helping in the reduced neutralization to these antibodies.

**Table 4.** Docking of S-RBD of spike, Delta and Omicron against three monoclonal antibodies.

Antibodies	Spike protein (PDB)	Dock scores	
		pyDockWEB	HDOCK
Casirivimab	SARS-CoV-2 (6XDG)	-125.996	-275.49
	Delta (7W92)	-107.493	-181.94
	Omicron (7T9K)	-90.285	-224.4
Bamlanivimab	SARS-CoV-2 (7KMI)	-121.918	-407.74
	Delta (7W92)	-94.374	-241.61
	Omicron (7T9K)	-102.161	-332.91
Etesevimab	SARS-CoV-2 (7F7E)	-123.423	-263.07
	Delta (7W92)	-108.941	-183.8
	Omicron (7T9K)	-102.213	-180.94

S-RBD: Spike-receptor binding domain.

## Discussion

The current study signifies the importance of mutations in the S-RBD region of Omicron in the enhancement of its binding affinity with the human ACE2 receptor. Omicron, a recent novel variant of SARS-CoV-2 was first detected in South Africa and has spread globally [16]. Scientists were perplexed by the presence of the number of mutations in the virus. Out of total 42 mutations, 15 mutations were present in the S-RBD region of the virus, which binds with the host ACE2 receptor to promote its virulence [29]. Interaction between the critical residues dictates the strength of binding between the complex, and the variability in the binding affinity depends on the type and nature of amino acids. Thus, it was hypothesized that the mutations present in the S-RBD region of Omicron would provide an evolutionary advantage to the virus in enhancing its binding with the receptor and thus its transmissibility and infectivity. To confirm the above hypothesis, *in silico* site-directed mutations were created on the crystal structure of S-RBD of SARS-CoV-2 (PDB 6M17_A) in accordance with the mutations present on the S-RBD region of Omicron. (Table 1). Docking analysis between the mutated S-RBD structure of Omicron and ACE2 receptor (PDB 6M17_B) was conducted to decipher the effect of mutations on the binding affinity of the virus with the receptor. Similarly, docking analysis of the S-RBD region of wild-type SARS-CoV-2 with the ACE2 receptor was also conducted to compare the change in binding affinity. Also, site-directed mutations in accordance with the mutations present in the S-RBD region of the Delta variant (Table 1) were conducted and the resulting structure was docked with ACE2 receptor to understand whether Omicron has a higher binding affinity with ACE2 compared with the Delta variant (Table 2). The docking results signified that the S-RBD region of Omicron binds with higher affinity to the ACE2 receptor, followed by Delta and wild-type SARS-CoV-2. This result aligns with the previous studies which also suggested that Omicron has a higher binding affinity with the ACE2 compared with Delta and wild-type SARS-CoV-2 [34,35].

To decipher the importance of the mutations on the S-RBD region of the virus, 3Å interacting residues between the docked complex were analyzed to reveal whether mutated residues are involved in the binding or not. The result indicated that four mutated residues in the S-RBD region of Omicron (K417N, Q493R, G496S and N501Y) were involved in the binding with the ACE2 receptor (Table 3). Thus, it might be speculated that these mutations may confer an advantage to the virus in mediating the enhanced binding and thus its transmissibility. Previous studies have also found that mutations Q493R, G496S and N501Y form new hydrogen bonds and salt bridges with the ACE2 receptor, which might govern its increased binding affinity. Also, mutation K417N have been found to increase the binding affinity of the S-RBD with the spike protein when present in conjunction with other mutations like E484K and N501Y [36,37]. Analyses of 3Å interacting residues between the docked complex of S-RBD of Delta and ACE2 receptor didn't show any mutated residues binding with the receptor (Table 3), although these residues have been found to be involved in impaired neutralization to antibodies and in antigenic escape [38].

mAbs are widely used in the treatment of SARS-CoV-2 and have shown enhanced neutralizing activity [39]. To computationally predict the binding affinity and thus the neutralizing capacity of these antibodies against the S-RBD region of the SARS-CoV-2, Delta and Omicron, three potent mAbs (casirivimab, bamlanivimab and etesevimab) were docked against the S-RBD region of the virus. The result suggested that the variants mediate reduced binding affinity and thus reduced neutralization capacity against the mAbs compared with wild-type S-RBD of SARS-CoV-2 (Table 4, Figure 2 & Supplementary Figure 1). This also implicates those mutations present in the S-RBD region of the variants may confer an evolutionary advantage to the virus in its decreased neutralization to the mAbs. Our current data aligns with the previous findings that variants show decreased neutralization capacity to the mAbs compared with wild-type SARS-CoV-2 [40,41]. To address the issue of decreased antibody neutralization against the SARS-CoV-2 and VOC strains, mAbs cocktail which binds to different epitopes on the spike protein or to the highly conserved regions on the virus not amendable to mutations, such as non-RBM region of the RBD, could be a useful strategy to overcame the virus resistance to individual mAbs [42,43].

To summarize the above findings, the current *in silico* analyses propose that due to the number of mutations on the S-RBD region of Omicron, it confers enhanced binding affinity with the ACE2 receptor compared with S-RBD of Delta and wild-type SARS-CoV-2. Specifically, it also signifies the importance of four mutated residues present on the S-RBD of the Omicron in enhancing its binding affinity with the ACE2 receptor. Furthermore, docking of S-RBD of the variants with the three approved mAbs showed reduced binding affinity to the mAbs compared with wild-type SARS-CoV-2, which suggests decreased neutralization capacity of the mAb against the variants. However, these *in silico* findings have their own limitations and thus *in vitro* studies should be carried out to replicate and confirm the above findings.

## Conclusion

The interaction of the virus with the host plays a crucial role in its entry into the host cell and serves as a factor in the rate of infection. The higher affinity between SARS-CoV-2 variants and host can initially suggest the knowledge of its spread and infectivity. We have previously observed that the other identified SARS-CoV-2 VOCs have a strong binding affinity of their S-RBD with the host ACE2 receptor when compared with the S-RBD of SARS CoV-2, as well as evidence suggests their high pathogenic nature [15]. The mutations in SARS-CoV-2 have led to significant changes in variants transmission and resistance mechanisms against the host immune system or some extent against anti-SARS-CoV-2 vaccines. The mutations in the Omicron variant differ from other VOCs and their numbers are very high. It contains fifteen mutations in spike RBD itself as compared with two or three mutations in RBD of other VOCs. Therefore, we proposed to study the binding affinity of the novel Omicron variant along with the Delta variant with the host ACE2. We performed the *in silico* mutations of all 15 residues in wild-type SARS-CoV-2 spike RBD for Omicron and two mutations for Delta to perform our study. Our findings strongly indicate that Omicron S-RBD has an enhanced affinity with the ACE2 receptor than the wild-type and Delta SARS-CoV-2, which may lead to increased infectivity and spread of the virus. It also highlights the importance of four mutated residues in the S-RBD region of the Omicron (K417N, Q493R, G496S and N501Y) being involved in the interaction with ACE2 receptor closely within the 3Å region. It also suggests that these residues may have a significant role in the S-RBD and ACE2 interaction in the Omicron and the future direction would be to analyze these four residues by *in vitro* site-directed mutagenesis to confirm their role in the binding affinity of the Omicron S-RBD with the ACE2. We also determined that mAbs designed against SARS-CoV-2 spike protein showed decreased neutralizing activity with the other VOC owing to their decrease in binding affinity. The mutations affect mainly the enhanced binding of VOC with the ACE2 receptor of host cells as well as reduced binding of mAbs to the variants.

**Summary points**

- The current study signifies the importance of mutated residues on the S-receptor binding domain (S-RBD) of Omicron in enhancing the binding affinity with ACE2.
- *In silico* site-directed mutations were created on the crystal structure of S-RBD of wild-type SARS-CoV-2 in accordance with the mutations present on the S-RBD of Omicron and Delta and the resulting structure was docked against the ACE2 receptor.
- The result indicated S-RBD of Omicron showed higher binding affinity to ACE2 receptor compared with the S-RBD of Delta and wild-type SARS-CoV-2. 3Å interacting residues between the complex signified four mutated residues in S-RBD of Omicron (K417N, Q493R, G496S and N501Y) involved in the binding with the ACE2 receptor.
- Docking of three monoclonal antibodies (casirivimab, bamlanivimab and etesevimab) against the S-RBD of wild-type, Delta and Omicron suggested that variants showed reduced binding affinity and thus reduced neutralization capacity of the variants to the antibodies compared with wild-type SARS-CoV-2.
