# Supplementary material for: Structural analysis of spike proteins from SARS-CoV-2 variants of concern highlighting their functional alterations
Source: Future Virol. 2022 Aug 2:10.2217/fvl-2022-0003. doi: 10.2217/fvl-2022-0003 (PMC9345306; doi:10.2217/fvl-2022-0003)
Supplement: Supplementary file 1 [file Supplementary-figure.docx]

**Supplementary Data**


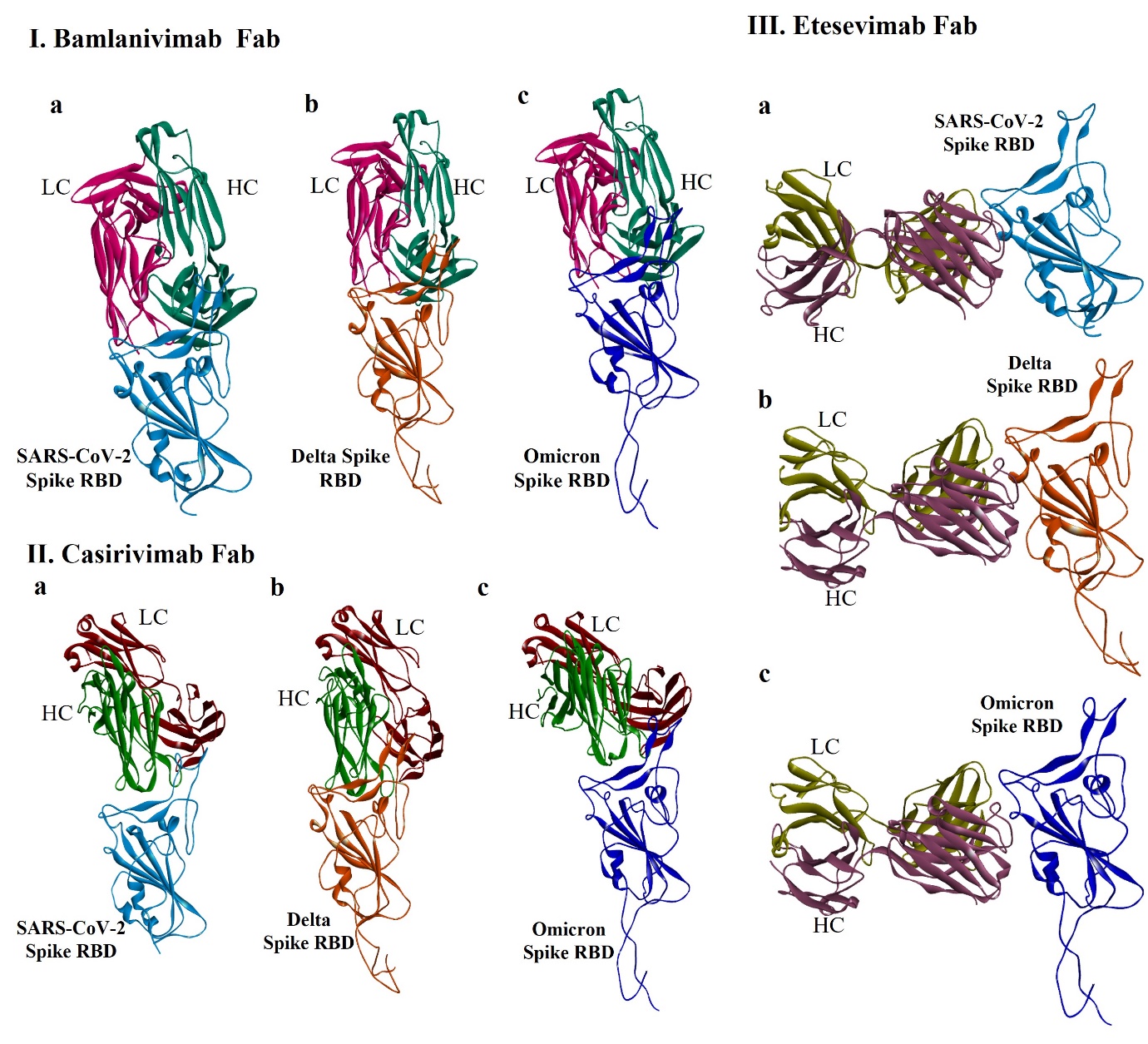


**Supplementary Figure S1:** Docking complex of S-RBD of SARS-CoV-2, Delta and Omicron with mAbs: I.) Bamlanivimab Fab (HC&LC) against S-RBD of a.) SARS-CoV-2, b.) Delta, and c.) Omicron; II) HDOCK analysis of Casirivimab Fab (HC&LC) against S-RBD of a.) SARS-CoV-2, b.) Delta, and c.) Omicron; III) HDOCK analysis of Etesevimab Fab (HC& LC) against S-RBD of a.) SARS-CoV-2, b.) Delta, and c) Omicron. SARS-CoV-2 Spike RBD is shown in light blue; Delta Spike RBD is shown in orange; Omicron Spike RBD is shown in dark blue; Bamlanivimab Fab heavy chain is shown in dark green; light chain is shown in light red; Casirivimab Fab heavy chain is shown in light green; light chain is shown in maroon; Etesevimab Fab heavy chain is shown in violet; light chain is shown in dark yellow. RBD: Receptor binding domain; SARS-CoV-2: severe acute respiratory syndrome coronavirus 2; HC: Heavy chain; LC: Light chain; Fab: Fragment antigen-binding region.
